# Plasma cholesterol in Alzheimer’s disease and frontotemporal dementia

**DOI:** 10.1515/tnsci-2020-0098

**Published:** 2020-05-18

**Authors:** Pan Wang, Huihong Zhang, Yan Wang, Miao Zhang, Yuying Zhou

**Affiliations:** Department of Neurology, Tianjin Huanhu Hospital, Nankai University, Tianjin, 300350, China

**Keywords:** Alzheimer’s disease, apolipoprotein E gene, Frontotemporal dementia, lipid metabolism

## Abstract

**Background:**

The relationship between the apolipoprotein E (*APOE*)-*ε4* allele, triglyceride (TG) level, and cholesterol level and an increased risk of developing Alzheimer’s disease (AD) has been well established, but their relationship with behavioral-variant frontotemporal dementia (bvFTD) is not well-known.

**Methodology:**

The levels of TGs, total cholesterol (TC), low-density lipoprotein (LDL), and high-density lipoprotein were measured in bvFTD and AD patients and in normal controls (NCs). DNA was extracted, and *APOE* was genotyped.

**Results:**

The *APOE-ε4* allele frequency was higher in the AD group than in the NC group, but no difference was found between the AD and the bvFTD groups. The bvFTD group had higher LDL than the AD group, and significant differences were also found for the cholesterol level in the dementia groups compared with the NC group. Elevated LDL level was positively correlated with appetite and eating score in the bvFTD group. Compared with the AD patients and NCs without the *APOE-ε4* allele, those with the *APOE-ε4* allele had higher TC, but its correlation with the bvFTD group was absent.

**Conclusions:**

The bvFTD and the AD groups had higher cholesterol levels. The *APOE-ε4* allele and eating behavior might modify lipid metabolism in dementia. TG and cholesterol analyses may offer a new opportunity for targeted treatments.

## 1 Introduction

Alzheimer’s disease (AD) and frontotemporal degeneration (FTD), especially behavioral-variant frontotemporal dementia (bvFTD), are the two kinds of major degenerative dementia, and they are commonly misdiagnosed. There are both differences and similarities between them. The pathological mechanism of AD includes senile plaque formed with amyloid beta (Aβ) deposition and neurofibrillary tangles (NFTs) made of phosphorylated Tau (pTau). Tau misfolding and aggregation into beta-pleated sheets, containing oligomers and fibrils, occur in FTD [1]. The process of AD and FTD pathology results in loss of microtubule-binding function and formation of cytosolic tau inclusions. Clinically, the characteristic changes of AD include the loss of cortical neurons in the temporal and/or parietal lobes and memory loss, and those of bvFTD include frontal and/or temporal lobe pathology and behavioral abnormalities [2]. Additionally, the similarities between them lead to an overlap in clinical presentations, Tau pathology, and risk factors. The common risk factors include less education, smoking, obesity, lifestyle factors, and high serum cholesterol level.

Cholesterol is vital to the neuronal structure and function, and its disturbance has been shown to play potentially important roles in dementia. Initially, the main link between cholesterol and AD was its impact on atherosclerosis and cardiovascular disease [3], including stroke, cerebral amyloid angiopathy, small vessel disease, lacunes, perivascular spaces (PVS), white matter hyperintensity, and so on. While it is clear that high cholesterol induces vascular disease, alterations in cholesterol have also been linked to the pathological features of AD. Cholesterol plays potentially important roles in the synthesis, deposition, and clearance of Aβ [4], which is the main pathological basis of AD. The apolipoprotein E (*APOE*)*-ε4* allele is a strong risk gene for AD, and its interaction between cholesterol and AD has become a hot topic. In contrast to AD, hypercholesterolemia has not been implicated in FTD, and few reports about cholesterol have been found. Actually, there is significant loss of brain tissue with concomitant loss of lipids, and behavioral abnormalities, especially diet and eating behaviors, may also contribute to the changed cholesterol level [5].

The relationship between cholesterol and different dementia is complex. The lipid levels may be influenced in a variety of ways in AD and FTD, but the difference in lipid levels between these diseases is unclear. The relationship of the *APOE-ε4* allele to FTD is also a matter of dispute. Some studies have shown a link between the *APOE-ε4* allele and FTD [6,7], but other studies have shown that the *APOE-ε4* allele did not increase the risk of FTD [8,9]. Furthermore, the relationship of the *APOE-ε4* allele with lipid levels in FTD patients is unclear. Based on the above gaps in knowledge, we investigated the difference in lipid levels among the groups of AD, bvFTD, and normal controls (NCs) in the present study. We also discuss the differences in the *APOE* genotype and whether they may influence lipid levels in AD and bvFTD patients.

## 2 Methods

### 2.1 Subjects

Ninety-three patients with dementia (63 AD and 30 bvFTD) were recruited from the cognitive impairment clinic of Tianjin Huanhu Hospital. All assessments, including neurologic examination, routine laboratory tests (especially vitamin B12 level, thyroid hormone level, syphilis, and HIV serology), and cognitive function tests, were done by trained doctors. To ensure the accuracy of the diagnosis, more than half of the dementia patients (including the patients with bvFTD, definite diagnosis, and atypical AD) underwent ^C^11-PIB-PET and/or FDG-PET. All patients met the core clinical criteria of the new diagnostic criteria for probable bvFTD or AD [10,11,12], and clinical diagnosis was made by consensus between the neurologist and neuroradiologist. The criteria for exclusion from the study included significant neurological or psychiatric illness that could influence cognitive functions as well as significant unstable systemic illness or organ failure. Each subject was right-handed and underwent a battery of neuropsychological tests: the Mini-Mental State Examination (MMSE), Montreal Cognitive Assessment (MoCA), Neuropsychiatric Inventory (NPI), and Activities of Daily Living (ADL) scale. In addition, 33 age- and sex-matched healthy NCs without neurological or psychiatric disorders were included for comparison. None of the subjects used either statins or other lipid-modifying agents. All subjects were scanned using a single 3-T MRI scanner. To avoid the disturbance of cerebrovascular pathology in this study, the subjects with a high burden of vessel disease [13], including the subjects with Fazekas scale 2 and 3, multiple lacunar infarction, PVS > 11, and so on, based on STandards for Reporting Vascular changes on nEuroimaging [14] were excluded through magnetic resonance imaging.


**Ethical approval:** The research related to human use has been complied with all the relevant national regulations and institutional policies, is in accordance with the tenets of the Declaration of Helsinki, and has been approved by the Medical Ethics Committee of Tianjin Huanhu Hospital.
**Informed consent:** Informed consent has been obtained from all individuals included in this study or their legal guardians (spouses or children).

### 2.2 *APOE* genotyping

The blood samples were collected in tubes containing ethylenediaminetetraacetic acid as anticoagulant, and DNA was isolated from blood cell nuclei. *APOE* genotypes were determined by restriction enzyme digestion according to the method of Wenham *et al*. [15].

### 2.3 Measurement of cholesterol levels

Collection of blood samples was done within 3 days after enrollment. Blood samples were collected after an overnight fast of >12 h. The concentrations of triglycerides (TGs), total cholesterol (TC), and high-density lipoprotein (HDL) were measured using an automatic chemical analyzer (Beckman AU5800, USA) [16,17]. Low-density lipoprotein (LDL) levels were calculated using the Friedewald formula: LDL = [Cholesterol − (Triglycerides/2.2)] − HDL [18,19].

### 2.4 Statistical analysis

Data were analyzed using SPSS statistics (version 19.0) and are presented as mean ± standard deviation or proportions. The Kolmogorov–Smirnov test was run to determine the suitability of variables for parametric analysis. Analysis of variance, followed by Tukey’s *post hoc* test, was used to explore the main effects of the variables age, TGs, TC, and LDL (*P* < 0.05 was regarded as significant) on the groups (NCs, bvFTD, and AD). MMSE, MoCA, ADL, and NPI scores and HDL levels were analyzed using the Kruskal–Wallis test followed by the *post hoc* Mann–Whitney test corrected for multiple comparisons (*P* < 0.01 was regarded as significant).

Allele frequencies were assessed by counting alleles and calculating proportions. AD, bvFTD, and NC groups were stratified according to the presence or absence of the *APOE-ε4* allele; the number of *APOE-ε2*, *ε3*, and *ε4* alleles; and *APOE* genotype (*APOE-ε2/ε2*, *APOE-ε2/ε3*, *APOE-ε2/ε4*, *APOE-ε3/ε3*, *APOE-ε3/ε4*, *APOE-ε4/ε4*). The *χ* ^2^ test and the *post hoc* Fisher exact test (*P* < 0.05 was regarded as significant) were applied to compare allele frequencies and sex among them. To investigate the relationship between lipid levels and psychological and behavioral abnormalities, we also explored Pearson’s correlations between lipid levels and the subscale scores (appetite and eating change) of the NPI. To clarify the potential effects of the *APOE* genotype, groups were additionally divided into two subgroups according to the presence or absence of at least one *APOE-ε4* allele.

## 3 Results

The demographic and clinical characteristics of the AD, bvFTD, and NC groups are summarized in Table 1. No significant differences in sex or age were detected among the AD, bvFTD, and NC groups. Compared with NCs, the MMSE and MoCA scores were lower and the ADL score was higher in the AD and bvFTD groups. But no difference was found between the AD and bvFTD groups (Table 1). The bvFTD patients had a higher NPI score than the AD patients (AD: 9.1 ± 1.3 vs bvFTD: 20.2 ± 3.0, *P* < 0.001). Within the subscale scores of the NPI, the scores of agitation/aggression, anxiety, apathy, disinhibition, irritability, aberrant motor behavior, appetite, and eating change were significantly higher in the bvFTD patients than in the AD patients (*P* < 0.05).

**Table 1:** Demographic characteristics and cognitive scores for the AD, bvFTD, and NCs

	AD (*n* = 63)	bvFTD (*n* = 30)	NCs (*n* = 33)	*P*
Sex (M/F)	63 (30/33)	30 (8/22)	33 (22/11)	0.11
Age (years)	66.3 ± 9.6	64.5 ± 7.7	66.0 ± 8.7	0.64
MMSE	16.2 ± 6.6	16.7 ± 8.7	28.7 ± 1.2	<0.001^a,b^
MoCA	10.8 ± 6.2	10.2 ± 6.8	26.8 ± 1.6	<0.001^a,b^
ADL	28.7 ± 11.0	31.1 ± 10.5	20.0 ± 0.0	<0.001^a,b^
NPI	9.1 ± 1.3	20.2 ± 3.0	0.3 ± 0.2	<0.001^a,b,c^

Significant differences in demographic or clinical data between groups are denoted as follows: ^a,b,c^ *P* < 0.05.

^a^AD vs NCs; ^b^bvFTD vs NCs; and ^c^AD vs bvFTD.

AD: Alzheimer’s disease; bvFTD: behavioral-variant frontotemporal dementia; NCs: normal controls; MMSE: Mini-Mental State Examination; MoCA: Montreal Cognitive Assessment; ADL: activities of daily living; NPI: neuropsychiatric inventory.

The distribution of *APOE* genotypes and the corresponding allele frequencies of the groups are shown in Table 2. The allele frequencies were 3.2% for *ε2*, 69.8% for *ε3*, and 27.0% for *ε4* in the AD group and 6.7% for *ε2,* 75% for *ε3*, and 18.3% for *ε4* in the bvFTD group. Compared with NCs, the allelic frequency of *APOE-ε4* was higher in the AD and bvFTD groups, and the difference between the AD and NC groups was significant (*P* < 0.05).

**Table 2:** Genetic data in the AD, bvFTD, and NC groups

	AD (*n* = 63)	bvFTD (*n* = 30)	NCs (*n* = 33)	*P*
*APOE* genotype: number of patients (%)				
*APOE-ε2/ε2*	0 (0)	0 (0)	0 (0)	
*APOE-ε2/ε3*	0 (0)	0 (0)	4 (12.1)	
*APOE-ε2/ε4*	4 (6.3)	4 (13.3)	0 (0)	
*APOE-ε3/ε3*	35 (55.6)	20 (66.7)	21 (63.6)	
*APOE-ε3/ε4*	18 (28.6)	5 (16.7)	8 (24.2)	
*APOE-ε4/ε4*	6 (9.5)	1 (3.3)	0 (0)	
*APOE* allele frequencies				
*APOE-ε2*, %	3.2	6.7	6.1	0.49
*APOE-ε3*, %	69.8	75	81.8	0.195
*APOE-ε4*, %	27	18.3	12.1	0.047*

*NCs vs AD. AD: Alzheimer’s disease; bvFTD: behavioral-variant frontotemporal dementia; NCs: normal controls.

No difference was found across the groups in the TG level (*P* = 0.172). In contrast, TC, HDL, and LDL levels differed across the groups, with the AD and bvFTD groups exhibiting a higher level than the NC group (*P* < 0.001; Table 3). No difference was found between the AD and bvFTD groups in the TC or HDL level (*P* > 0.05), but the bvFTD group exhibited a significantly higher LDL cholesterol level compared with the AD group (*P* = 0.04). We further analyzed the relationship between lipid levels and behavioral and psychological symptoms. The LDL level was positively correlated with the scores of appetite and eating in the bvFTD group (*r* = 0.375, *P* = 0.04).

**Table 3:** Lipid levels in the AD, bvFTD, and NC groups

	AD (*n* = 63)	bvFTD (*n* = 30)	NCs (*n* = 33)	*P*
TG (mmol/L)	1.26 ± 0.54	1.54 ± 0.98	1.37 ± 0.54	0.172
TC (mmol/L)	5.50 ± 0.98	5.63 ± 0.94	4.5 ± 0.71	<0.001^a,b^
HDL (mmol/L)	1.50 ± 0.40	1.48 ± 0.40	1.18 ± 0.23	<0.001^a,b^
LDL (mmol/L)	3.06 ± 0.59	3.34 ± 0.74	2.41 ± 0.60	0.001^a,b,c^

AD: Alzheimer’s disease; bvFTD: behavioral-variant frontotemporal dementia; NCs: normal controls; TG: triglyceride; TC: total cholesterol; HDL: high-density lipoprotein; LDL: low-density lipoprotein.

*Post hoc* comparison of significant group differences: ^a,b,c^ *P* < 0.05.

^a^AD vs NCs; ^b^bvFTD vs NCs; and ^c^AD vs bvFTD.

Then, the three groups were dichotomized according to the presence of an *APOE-ε4* allele. Compared with the subjects without an *APOE-ε4* allele, the TC level was significantly higher in those with any *ε4* allele within the AD group (*P* < 0.05) (Table 4), and the TC and LDL levels were also higher in those with any ε4 allele within the NC group (*P* < 0.05) (Table 5). No difference was found between the genotype groups in the bvFTD group (Table 6).

**Table 4:** *APOE-ε4* allele and lipid levels in AD patients

	*APOE-ε4*+ (*n* = 28)	*APOE-ε4*− (*n* = 35)	*P*
TG (mmol/L)	1.3 ± 0.1	1.3 ± 0.1	0.992
TC (mmol/L)	5.9 ± 0.2	5.2 ± 0.1	0.01
HDL (mmol/L)	1.6 ± 0.1	1.4 ± 0.6	0.074
LDL (mmol/L)	3.2 ± 0.1	2.9 ± 0.1	0.073

AD: Alzheimer’s disease; TG: triglyceride; TC: total cholesterol; HDL: high-density lipoprotein; LDL: low-density lipoprotein.

**Table 5:** *APOE-ε4* allele and lipid levels in the NC group

	*APOE-ε4*+ (*n* = 8)	*APOE-ε4*− (*n* = 25)	*P*
TC (mmol/L)	5.0 ± 0.4	4.4 ± 0.7	0.018
TG (mmol/L)	1.5 ± 0.3	1.3 ± 0.6	0.525
HDL (mmol/L)	1.2 ± 0.3	1.2 ± 0.2	0.698
LDL (mmol/L)	2.9 ± 0.1	2.3 ± 0.6	<0.001

NC: normal control; TC: total cholesterol; TG: triglyceride; HDL: high-density lipoprotein; LDL: low-density lipoprotein.

**Table 6:** *APOE-ε4* allele and lipid levels in bvFTD patients

	*APOE-ε4*+ (*n* = 10)	*APOE-ε4*− (*n* = 20)	*P*
TC	5.9 ± 0.4	5.5 ± 0.2	0.261
TG	1.7 ± 0.4	1.4 ± 0.2	0.513
HDL	1.4 ± 0.1	1.5 ± 0.1	0.627
LDL	3.6 ± 0.3	3.2 ± 0.1	0.173

bvFTD: behavioral-variant frontotemporal dementia; TC: total cholesterol; TG: triglyceride; HDL: high-density lipoprotein; LDL: low-density lipoprotein.

## 4 Discussion

This study examined lipid levels across the AD and bvFTD groups to investigate the relationship between lipid levels, *APOE* genotype, and eating behavior. We uncovered changes in TC and LDL cholesterol levels in the AD and bvFTD patients, more serious mental behavioral abnormalities in bvFTD patients, and an increased frequency of the *ε4* allele in AD patients. When we further analyzed the influence of behavioral and psychological symptoms or *APOE* genotype on lipid levels, we found that the *APOE-ε4* genotype might influence the level of LDL in the AD and NC groups, and abnormal eating behaviors might affect LDL in bvFTD patients.

### 4.1 The frequency of the ε4 allele in AD and bvFTD

The prevalence of the *APOE-ε4* allele was higher in AD patients than in bvFTD patients and NCs, but the difference between the bvFTD patients and NCs did not reach statistical significance in our study. A higher number of *APOE-ε4* alleles are now considered to be the most important risk factors for AD. However, the published data on *APOE* allele frequencies in FTD are conflicting. Some studies reported a higher *ε4* allele frequency and found a significantly increased risk for the *ε4* allele in patients with FTD than in controls [20]. However, several studies and our findings have not replicated this result. An autopsy study of various dementias found that the *APOE-ε4* allele frequency was significantly elevated in AD but was comparable to the control frequency in FTD [21]. Another meta-analysis also found no significant result with the *ε4* allele in FTD [22]. These differences in FTD were attributed to different ethnicities and selection effects, but we solely focused on bvFTD, a subtype of FTD, in Chinese patients. Another reason might be the relatively small sample size, and the larger case–control study only included 94 FTD patients [22]. Finally, all the dementia diagnoses were made clinically without neuropathological confirmation, although with the help of ^11^C-PIB-PET and/or FDG-PET. The misdiagnoses might have reduced the power of the test.

### 4.2 Lipid levels in AD and bvFTD

It is well-known that the main component of senile plaque is Aβ, the main pathological basis of AD. Aβ is produced from the dual cleavage of amyloid precursor protein (APP) by BACE1 and γ-secretase. APP, β-secretase, and γ-secretase are located in lipid rafts, a kind of membrane-bound cholesterol, where APP metabolism occurs. Experiments in cell cultures indicated that cholesterol influences APP-secretase activity and that its accumulation in neurons might accelerate the decomposition of APPs into amyloidogenic components [23]. A growing number of studies have found that high cholesterol levels in the brain play an important role in the process of Aβ-induced AD [24]. In addition to high cholesterol in the brain, animal and human experiments have reported a link between serum cholesterol and Aβ deposition. For example, transgenic APP mice fed with a high cholesterol diet and hypercholesterolemic rabbits had accumulation of intracellular immunolabeled beta-amyloid protein in the brain [25,26]. At the same time, Reed and his colleagues found a direct relationship between cholesterol fractions in blood and amyloid deposition in the brain [27]. Another study also found significant positive associations of TC and LDL with the density of neuritic plaques, an AD hallmark [28]. In our study, higher levels of serum TC, HDL, and LDL were found in AD patients. However, it is important to recognize that CNS cholesterol is locally synthesized instead of coming from peripheral blood. The blood–brain barrier (BBB) separates cholesterol into the cerebral and extracerebral pools. In contrast to cholesterol itself, the side-chain oxidized metabolite 27-hydroxycholesterol (27OH) is able to pass the BBB and communicate between plasma cholesterol and the brain [29,30,31]. The level of 27OH in the circulation is proportional to the level of cholesterol, and there is a concentration-driven flux of 27OH from the circulation into the brain [29,32]. The accumulation of 27OH was found to be the most significant change in the sterol profile of the brains of patients with AD [33], and part of this accumulation is likely to have been affected by the level of cholesterol in the circulation. The high level of 27OH increases the formation of β-amyloid by antagonizing the suppressive effect of 24S-hydroxycholesterol [31,34] and suppresses the formation of activity-regulated cytoskeleton-associated protein by affecting the *N*-methyl-d-aspartate receptor [35]. Björkhem and his colleagues found that the flux of 27OH into the brain might be the link between hypercholesterolemia and AD [30,31]. In conclusion, higher cholesterol levels are associated with the further development of AD and represent an independent risk factor for AD [36].

Our study also uncovered high cholesterol levels in bvFTD patients, and the LDL level was correlated with the scores of appetite and eating. The changes in eating behavior are core symptoms of the diagnostic criteria for bvFTD, and abnormal eating behaviors are present in >60% of the patients, even >80% over the course of the disease [37]. The abnormal eating behaviors mainly include overeating, stuffing food into the mouth, and changes in food preferences (craving for carbohydrates) [38]. These eating behaviors are likely to contribute to high cholesterol levels. Recent studies revealed that eating abnormalities were partly related to hypothalamic degeneration and to potential disintegration of the network connections between the hypothalamus and reward pathways [38,39]. Therefore, the study of lipid metabolism might provide invaluable data for understanding the pathogenesis of bvFTD. Consistent with our results, another sample of Chinese bvFTD patients presented higher TC and LDL levels compared with NCs, and LDL level was associated with the diagnosis of FTD [40]. However, research from other countries found high TGs and high body mass index (BMI) in bvFTD, but not cholesterol levels [5,18]. Among the reasons involved in this variation, the first was the significant difference in tastes, diets, and perceptions from ethnic differences. Another reason was that lipid levels might be influenced not only by *APOE* genotype but also by the timing of lipid measurements in relationship to the stage of dementia [41]. High blood pressure and BMI have been associated with an increased risk of dementia for >10 years of follow-up [42], but there is a null or opposite relationship for <10 years [43]. The range of findings might hint at the fact that several years before dementia, onset blood pressure and BMI began to decline, possibly as a result of the ongoing neurodegenerative pathology, suggesting that the same may be true for TGs. Furthermore, large-sample and longitudinal research is required to determine the lipid levels in different diseases and stages. Overall, we suggest that adjusting eating behavior and dietary habits are possible targeting treatments that can modify eating behavior, metabolic abnormalities, and disease progression [44].

Pathological aggregation of the microtubule-associated protein Tau is a common feature of many neurodegenerative diseases, such as AD and bvFTD. The role of cholesterol in Tau phosphorylation or Tau proteostasis is not clear. The disturbance in cholesterol metabolism might impair the degradation of Tau aggregates [45]. The injection of Aβ42 into rat cortices caused a significant increase in hyperphosphorylation of Tau protein [46]. Some researchers argued that Aβ can induce Tau phosphorylation, and excess cholesterol may indirectly promote the production of NFTs. However, a new study implicated cholesterol as a dual upstream regulator of pTau and Aβ and suggested that reducing cholesterol potently decreases the neuronal pTau level through proteasomal upregulation and degradation of pTau in an APP- and Aβ-independent manner [47].

### 4.3 Effects of *APOE* genotype on lipid levels

Many candidate genes were considered to be related to lipid metabolism, and among them, the *APOE* genotype was the major source of genetically determined variation in lipid metabolism. For the subjects who carried the *APOE-ε4* allele, the levels of TC and/or LDL were higher than in those without the *APOE-ε4* allele in the AD and NC groups. Our findings reflected previous research that *APOE-ε4* carriers are associated with increased concentrations of serum TC and LDL [48]. APOE plays significant roles in binding the low-density lipoprotein receptor (LDLR) and the LDLR-related protein and in facilitating cellular uptake of lipoprotein particles [49]. The difference in the amino acid sequences of *ε2*, *ε3*, and *ε4* alleles results in different receptor-binding affinities. It could be hypothesized that the increase in TAG-rich lipoproteins of *APOE* in *ε4* carriers could increase the affinity to bind LDLR. Ultimately, this could reduce LDL uptake and increase circulating plasma cholesterol [50]. The presence of the *ε4* allele has been correlated with the risk of coronary heart disease [51] and with the risk of AD. This is attributable partly to the impact of *APOE* polymorphism on cholesterol metabolism. Compared with AD, bvFTD is a highly heterogeneous disease, and lipid metabolism is affected by many different factors. Our results show that changes in eating behavior might mediate changes in lipid levels and result in the lack of correlation between *APOE-ε4* and cholesterol level in bvFTD.

## 5 Conclusion

This study examined lipid levels across the AD and bvFTD groups to investigate the relationship between lipid levels, *APOE* genotype, and eating behavior. We found that the AD and bvFTD patients exhibited significantly higher cholesterol levels than the NCs. To further analyze the influencing factors of lipid levels, there was a significant interaction between cholesterol level and *APOE-ε4* in the AD and NC groups and between LDL level and changes in eating behavior in the bvFTD group. All of these findings offer opportunities for targeted treatments.
